# Coexpression Network Analysis-Based Identification of Critical Genes Differentiating between Latent and Active Tuberculosis

**DOI:** 10.1155/2022/2090560

**Published:** 2022-11-11

**Authors:** Liang Chen, Jie Hua, Xiaopu He

**Affiliations:** ^1^Department of Infectious Diseases, Nanjing Lishui People's Hospital, Zhongda Hospital Lishui Branch, Southeast University, Nanjing, China; ^2^Department of Gastroenterology, Liyang People's Hospital, Liyang Branch Hospital of Jiangsu Province Hospital, Nanjing, China; ^3^Department of Geriatric Gastroenterology, The First Affiliated Hospital with Nanjing Medical University, Nanjing, China

## Abstract

**Methods:**

Three Gene Expression Omnibus (GEO) microarray datasets (GSE19491, GSE98461, and GSE152532) were downloaded, with GSE19491 and GSE98461 then being merged to form a training dataset. Hub genes capable of differentiating between ATB and LTBI were then identified through differential expression analyses and a WGCNA analysis of this training dataset. Receiver operating characteristic (ROC) curves were then used to gauge to the diagnostic accuracy of these hub genes in the test dataset (GSE152532). Gene expression-based immune cell infiltration and the relationship between such infiltration and hub gene expression were further assessed via a single-sample gene set enrichment analysis (ssGSEA).

**Results:**

In total, 485 differentially expressed genes were analyzed, with the WGCNA approach yielding 8 coexpression models. Of these, the black module was the most closely correlated with ATB. In total, five hub genes (FBXO6, ATF3, GBP1, GBP4, and GBP5) were identified as potential biomarkers associated with LTBI progression to ATB based on a combination of differential expression and LASSO analyses. The area under the ROC curve values for these five genes ranged from 0.8 to 0.9 in the test dataset, and ssGSEA revealed the expression of these genes to be negatively correlated with lymphocyte activity but positively correlated with myeloid and inflammatory cell activity.

**Conclusion:**

The five hub genes identified in this study may play a novel role in tuberculosis-related immunopathology and offer value as novel biomarkers differentiating LTBI from ATB.

## 1. Introduction

Tuberculosis (TB) is a serious communicable disease caused by *Mycobacterium tuberculosis* (*Mtb*), ranking among the top 10 causes of death in the world as the deadliest pathogen-associated disease [1]. In 2019 alone, there were an estimated 10 million new TB diagnoses and 1.4 million deaths according to the Global Tuberculosis Report 2020 [2].

An estimated 25% of *Mtb-*infected individuals harbor an asymptomatic form of latent tuberculosis infection (LTBI), but 5-15% of these patients will ultimately progress to active TB (ATB) at some point in their lives [3, 4]. Clinical efforts to differentiate between LTBI and ATB remain challenging at an early stage, yet are critical to ensuring that these patients receive appropriate treatment to prevent the further spread of TB. Two of the most common strategies used to detect an individual's *Mtb* infection status are the cellular immunity-based tuberculin skin test (TST) and interferon-gamma release assay (IGRA) approaches, yet neither can reliably differentiate between LTBI and ATB patients [5, 6]. As such, it is vital that reliable biomarkers capable of distinguishing between individuals with latent and active TB be defined and validated.

The advent of microarrays and other high-throughput sequencing strategies has enabled the bioinformatics-based screening for biomarkers associated with particular disease states [7, 8]. Recent transcriptomic analyses have revealed distinct changes in circulating host leukocyte gene expression patterns as a function of the stage of *Mtb* infection [9–11]. Owing to heterogeneity among donors, sampling strategies, sequencing platforms, and analytical approaches, however, it can be challenging to establish reliable, clinically relevant data from individual analyses. As such, integrated bioinformatics strategies have been used to gain more comprehensive insight into the molecular pathogenesis of *Mtb* infection as a means of better defining biomarkers associated with different stages of disease. A weighted gene coexpression network analysis (WGCNA) is a systems biology approach that can be used to analyze patterns of gene connection across different models, describing interactions among genes and associated pathways rather than focusing on the identification of individual gene targets through a holistic assessment of gene set endogeneity and the relationship between these genes and phenotypes of interest [12]. As such, WGCNA methods can be readily leveraged to identify synergistic gene sets which may contain candidate biomarkers or therapeutic targets related to a particular disease state.

The present study was developed with the goal of using a WGCNA approach to identify key genes differentiating LTBI from ATB, with a further focus on the association between these genes and the infiltration of particular immune cell subsets as determined through a single-sample gene set enrichment analysis (ssGSEA).

## 2. Materials and Methods

### 2.1. Data Source

Data included in the present study were mRNA expression data derived from patient blood samples archived in the NCBI-GEO database (http://www.ncbi.nlm.nih.gov/geo). Downloaded datasets were those meeting the following criteria: (1) Patients were >15 years of age. (2) Samples were collected prior to the initiation of antimycobacterial treatment. (3) Patients were negative for severe autoimmunity, diabetes, malignant tumors, human immunodeficiency virus (HIV), immunosuppressive/immunomodulatory drug use, and coinfection with other pathogens and were not pregnant. Based on these three criteria, three datasets were selected for analysis (GSE19491, GSE98461, and GSE152532). The GSE19491 microarray dataset consisted of 69 and 89 whole-blood samples from LTBI and ATB patients, respectively. The GSE98461 microarray dataset consisted of 4 peripheral blood mononuclear cell (PBMC) samples each from LTBI and ATB patients. The GSE152532 microarray dataset consisted of 69 and 25 eligible whole-blood samples from LTBI and ATB patients, respectively. Differentially expressed genes (DEGs) were identified, and a WGCNA was performed by initially merging GSE19491 and GSE98461 into a training dataset, after which GSE152532 was used as the test dataset to validate identified hub genes. The training database included 93 male cases and 73 female cases, with a median age of 32.0 years (interquartile range [IQR]: 23.0-40.0 years). The test dataset concluded 64 male cases and 30 female cases, with a median age of 29.0 years (IQR: 19.0-34.3 years).

### 2.2. DEG Identification

The R (v 4.2.0) “GEOquery” and “limma” packages were used for the normalization and probe annotation of data in the training dataset, with DEGs being identified using the following criteria: adjusted *P* < 0.05, logFC > 1. Data were represented using a volcano plot, and the top 50 DEGs were assembled in the form of a heat map.

### 2.3. Functional Enrichment Analyses

The R “clusterprofiler” package was used to conduct GO and KEGG pathway enrichment analyses, with those terms/pathways yielding an adjusted *P* < 0.05 being considered significant. GO enrichment analyses were conducted for biological process (BP), molecular function (MF), and cellular component (CC) terms.

### 2.4. WGCNA Construction

Expression profile data from the training dataset were used to construct a WGCNA using the R “WGCNA” package, after which the genes exhibiting the top 25% absolute deviation from the median were selected for further study. Data integrity was analyzed using the “goodSampleGenes” function, while the “pickSoftThreshold” function was used for optimal soft threshold (*β*) selection. Matrix data were then used to establish an adjacency matrix, after which clustering was performed as a means of identifying modules based on the degrees of topological overlap. Module eigengene (ME) calculations were then performed, and similar modules were merged based on ME results, after which a hierarchical clustering dendrogram was established. Gene significance (GS) and module significance (MS) were calculated by combining modules and phenotypic data to determine clinical and gene-related significance and to assess correlations among models and modules. Module membership (MM) for individual genes was also assessed for each gene to assess module GS.

### 2.5. Hub Gene Screening and Validation

Candidate hub genes were selected via identifying those genes exhibiting the highest levels of intermodule connectivity. As biologically significant genes generally exhibit higher GS values, candidate hub gene identification was performed with the following criteria: |*GS*| > 0.20, |*MM*| > 0.80. Candidate hub genes and DEGs were then compared with the R “glmnet” package to identify overlap, with final hub genes being identified via a LASSO analysis.

Hub gene expression levels were compared between LTBI and ATB patients with box plots. The diagnostic utility of these hub genes when distinguishing between LTBI and ATB patients was then assessed using receiver operating characteristic (ROC) curves.

### 2.6. Immune Cell Infiltration and Hub Gene Correlation Analyses

Relative immune cell infiltration in training dataset samples was assessed with the ssGSEA algorithm, with differing levels of these cells being represented using violin plots. Spearman correlation analyses were used to compare the relationship between hub genes and infiltrating immune cells. The “ggplot2” package was used for result visualization.

## 3. Results

### 3.1. Identification and Functional Enrichment Analyses of DEGs Associated with LTBI and ATB Patients

**Figure 1:**
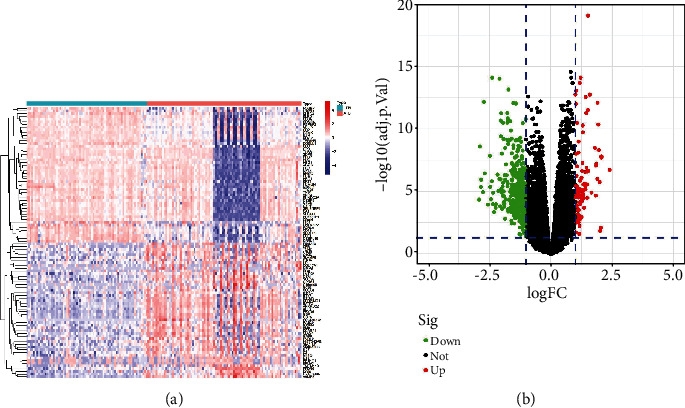
Gene expression profiling from the training set. (a) A heat map of the top 50 DEGs. Upregulated genes are shown in red, and downregulated genes are shown in blue. (b) A volcano plot of DEGs. Upregulated genes are shown in red, and downregulated genes are shown in green.

Initially, DEGs were identified by comparing microarray data between the ATB and LTBI patient cohorts in the training dataset. In total, 485 DEGs (91 upregulated, 394 downregulated) in the training set were identified when comparing LTBI and ATB patients (Figure 1).

**Figure 2:**
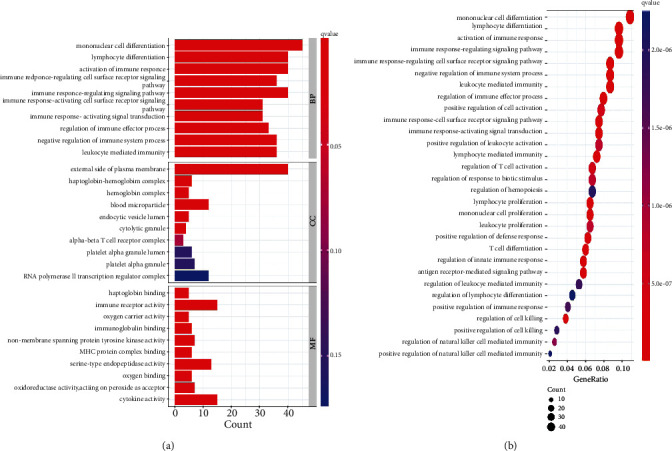
Functional enrichment analyses of DEGs from the training set. (a) GO enrichment analysis. (b) KEGG enrichment analysis.

Next, GO (Supplementary file 1) and KEGG (Supplementary file 2) analyses were conducted to explore the biological roles of these DEGs and associated signaling pathways underlying their potential role in the progression of LTBI to ATB. GO enrichment analyses revealed these DEGs to be enriched for terms relating to cellular differentiation (e.g., mononuclear cell differentiation and lymphocyte differentiation) and the regulation of the immune response (e.g., activation of the immune response, immune response-regulating cell surface receptor signaling pathway, and regulation of immune effector process) (Figure 2(a)). KEGG pathway analysis results were similar (Figure 2(b)). Together, these analyses suggested that immune and inflammation-associated processes are linked to LTBI progression to ATB.

### 3.2. WGCNA and Hub Gene Screening Analyses

**Figure 3:**
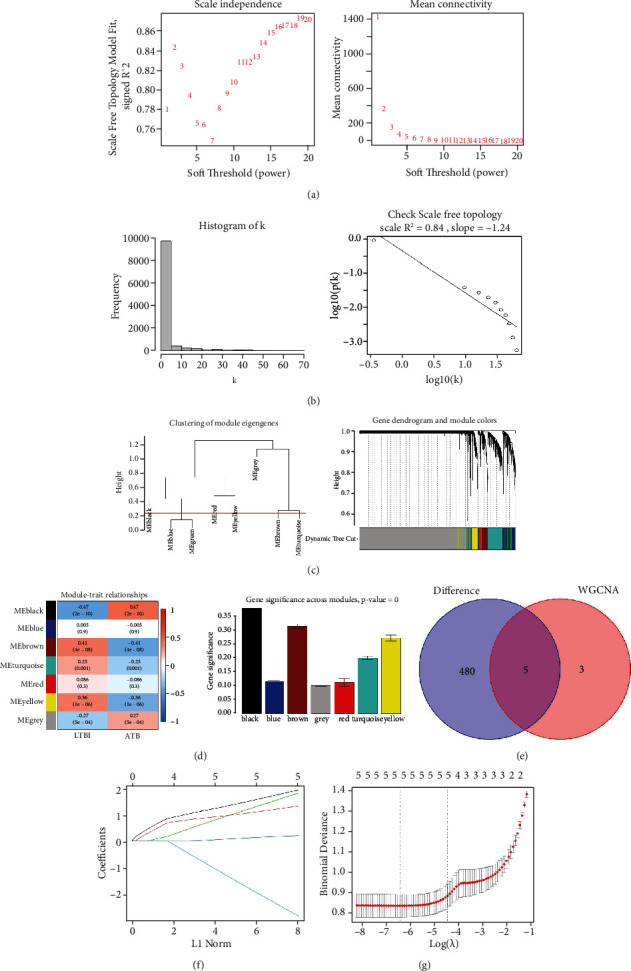
Construction of a weighted gene coexpression network analysis (WGCNA) and screening for hub genes. (a) Analysis of the scale-free fit index and the mean connectivity for various soft-thresholding powers (*β*). (b) Histogram of connectivity distributions and a check of the scale-free topology when *β* = 6. (c) Clustering of module eigengenes (the red line indicates a cut height of 0.25) and clustering dendrograms of genes based on a dissimilarity measure (1-TOM). (d) Module-trait associations were evaluated by correlations between module eigengenes and sample traits. Each cell contains the correlation coefficient and *P* value. (e) Venn diagram for the intersection between DEGs and the red module. (f) Partial likelihood deviance with changing of log (l) plotted via LASSO regression with 10-fold cross-validation. Dotted vertical lines were drawn at the optimal values using the minimum criteria (lambda.min) and 1 standard error of the minimum criteria (1-SE criteria). (g) The LASSO coefficient profiles for 3 hub genes in the 10-fold cross-validation.

To establish critical gene modules capable of differentiating between LTBI and ATB patients, a WGCNA approach was used to establish a coexpression network using the training dataset. In total, 7 modules were ultimately identified when assessing hub gene expression levels and diagnostic utility using a soft-thresholding power of 15 (scale-free *R*^2^ = 0.84, slope = −1.24) (Figures 3(a) and 3(b)) and cut height of 0.25 (Figure 3(c)). Correlations between ME values and sample traits were used to assess the potential relationships between these modules and clinical characteristics, revealing the black module to be the most closely linked to the progression of LTBI to ATB (*R* = 0.47, *P* = 2*e* − 10) (Figures 3(c) and 3(d)). The intersection of DEGs and this module revealed 5 overlapping genes (Figure 3(e)), with a LASSO analysis subsequently establishing these 5 genes as hub genes: FBXO6, ATF3, GBP1, GBP4, and GBP5 (Figures 3(f) and 3(g)).

### 3.3. Analyses of Hub Gene Expression Levels and Diagnostic Utility

**Figure 4:**
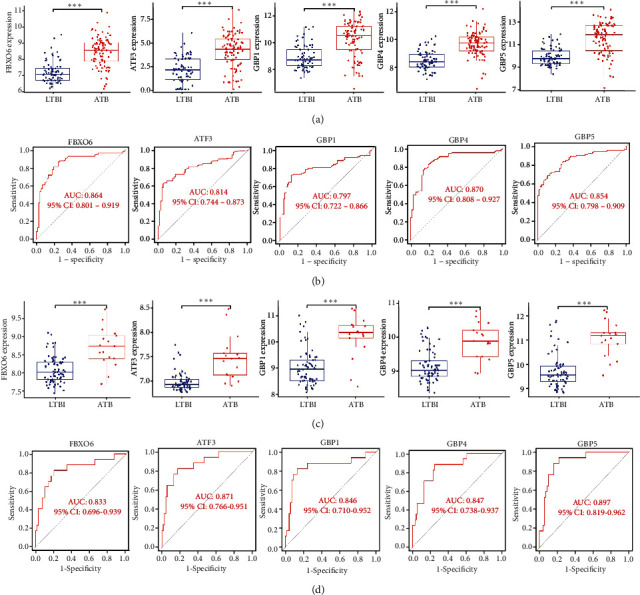
Validation of hub genes. (a) Validation of the expression levels of the hub genes performed using boxplots in the training set. (b) Validation of the diagnostic value of the indicated hub genes performed via ROC analyses in the training set. (c) Validation of the expression levels of the hub genes performed using boxplots in the test set. (d) Validation of the diagnostic value of the indicated hub genes performed via ROC analyses in the test set.

The expression of the identified hub genes was significantly increased in patients with ATB as compared to LTBI patients in the training dataset (Figure 4(a)), with similar findings also being observed in the test dataset (Figure 4(c)). ROC curve analyses of the training dataset revealed AUC values of 0.864 (95% CI: 0.801-0.919) for FBXO6, 0.814 (95% CI: 0.744-0.813) for ATF3, 0.797 (95% CI: 0.722-0.866) for GBP1, 0.870 (95% CI: 0.808-0.927) for GBP4, and 0.854 (95% CI: 0.798-0.909) for GBP5 (Figure 4(b)). Consistently, the AUC values for these 5 hub genes in the test dataset ranged from 0.800 to 0.900, indicating that they offer good diagnostic accuracy as a means of distinguishing between ATB and LTBI patients (Figure 4(d)).

### 3.4. Correlations between Hub Gene Expression and Immune Cell Infiltration

**Figure 5:**
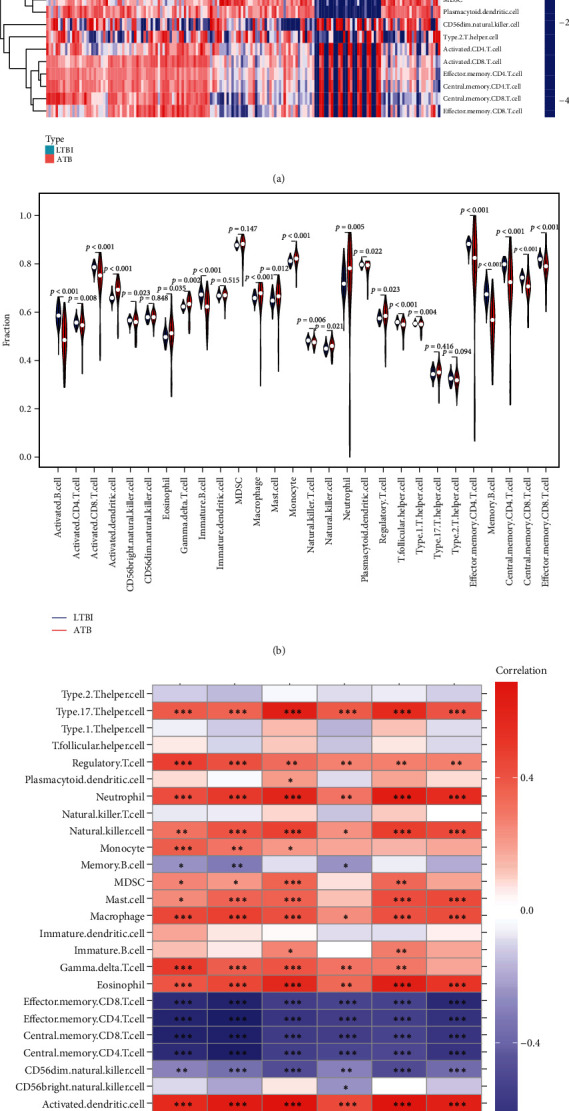
Analysis of the immune landscape associated with disease severity. Heat map (a) and violin plot (b) showing the distribution of immune cells in the ATB and LTBI groups. (c) The relationship between hub genes and immune cell infiltration.

Differences in immune cell infiltration were next compared between the ATB and LTBI patient cohorts using the ssGSEA algorithm, revealing significantly higher levels of myeloid and inflammatory cells (including monocytes, dendritic cells, macrophages, and neutrophils) in ATB patient samples, whereas lower lymphocyte levels (including activated B cells, memory B cells, activated CD8 T cells, memory CD8 T cells, activated CD4 T cells, and memory CD4 T cells) in LTBI patients (Figures 5(a) and 5(b)). Expression levels of these 5 hub genes were negatively correlated with lymphocyte levels and positively correlated with myeloid cell levels (Figure 5(c)).

## 4. Discussion

Transcriptomic analyses offer a robust approach to identifying and comprehensively evaluating biomarkers that can distinguish between patients suffering from LTBI or ATB. While several prior studies have demonstrated differences in host response-related gene expression profiles and the associated structure of these gene sets as a function of the stage of *Mtb* infection, most of these reports were based on individual cohort studies lacking corresponding functional analyses or clinical validation. In an effort to address this issue, the present study was conducted by using a systematic approach to analyze a combination of data from several datasets, ultimately leading to the identification of five central hub genes associated with the progression of LTBI to ATB. Relative to other bioinformatics techniques, WGCNA strategies exhibit robust advantages as they focus on analyses of the association between coexpression modules and clinical parameters of interest, providing more complete and reliable results that are more likely to be biologically meaningful [12].

Here, functional enrichment analyses revealed the DEGs identified when comparing the LTBI and ATB patient samples were primarily related to inflammation- and immunity-related pathways. Additional immune cell infiltration analyses suggested that in ATB patient samples, the levels of myeloid and inflammatory cells including DCs, monocytes, and neutrophils were enhanced relative to samples from LTBI patients, whereas T and B cell levels were reduced. Berry et al. [13] previously reported reductions in T cell- and B cell-specific gene signatures in a transcriptomic analysis of ATB patient samples, with flow cytometry analyses further supporting a decrease in both effector and central memory T cells in these patients consistent with the observed change in T cell-related gene expression. This is consistent with a growing body of evidence suggesting that the odds of developing ATB after infection are related to the monocyte/lymphocyte ratio [14, 15]. Joosten et al. [16] conducted an integrated analysis of eight independent TB microarray datasets and thereby revealed a significant association between TREM1 signaling pathway activity and myeloid cell activity in the context of ATB. Functionally, TREM1 expression promoted enhanced inflammatory responses driven by neutrophils and monocytes. Adaptive cellular immunity is the primary mechanism that controls chronic *Mtb* infections, contributing to the persistence of LTBI [17]. A diverse range of T cell subsets responding to many different *Mtb-*derived antigens is critical to the containment of these bacteria within macrophage-based granulomas. Specifically, CD4+ T cells secrete cytokine that can support macrophage-mediated *Mtb* control in addition to providing help that supports B cell-mediated antibody production and CD8+ T cell proliferation [18, 19]. In humans diagnosed with LTBI, *Mtb-*responsive MHC-I restricted CD8+ T cells have been detected in both the bronchoalveolar lavage fluid and blood [20]. Computer-based modeling efforts have highlighted that critical roles for multifunctional CD8+ T cells are barriers to the dissemination of *Mtb.* B cells are also important contributors to anti-TB immune responses, functioning within germinal centers to produce antibodies capable of modulating innate and adaptive immunity, enhancing the presentation of antigens to T cells, and producing cytokines that can support ongoing T cell responses [21, 22]. Both T cells and antibodies derived therefrom can influence granuloma formation and thus shape the progression of an *Mtb* infection. Accordingly, the inhibition of lymphocyte responses is generally linked with poorly controlled *Mtb* infection status and LTBI progression to ATB. In patients affected by ATB, host responses to the disseminated bacteria and associated tissue damage result in extensive inflammatory responses contributing to the proliferation of inflammatory cell populations including DCs, macrophages, monocytes, and neutrophils [23].

FBXO6 is a member of the F-box family of proteins, which serve as key components of the SKP1-Cullin1-F-box (SCF) E3 ligase that controls the proliferation, cell cycle progression, and survival of cells [24]. Du et al. [25] recently reported the ability of FBXO to upregulate type I IFN expression through a noncanonical mechanism independent of SCF E3 ligase activity whereby it can promote IRF3 ubiquitination and consequent degradation. While type I IFN signaling is critical in the context of many viral infections, it has been suggested to be deleterious in the regulation of *Mtb* and other bacterial infections [26, 27]. Chronic peripheral IFN response activation has also been shown to precede the onset of ATB [27]. While further work will be critical to fully establish the functional importance of IFN responses in the context of TB progression, both human cell- and mouse model-based data support the detrimental effect of type I IFN signaling on the induction of an effective immune response directed against TB. Transcriptomic analyses of blood samples from ATB patients have revealed IFN-inducible genes to form a major component of this disease-related gene signature, serving to promote inflammation and myeloid function while suppressing genes associated with T and B cell function [13, 28]. Guanylate-binding proteins (GBPs) are IFN-inducible GTPases that play key roles in shaping antibacterial immune responses and predict the progression of multiple different infectious diseases [29]. Within macrophages, intracellular *Mtb* can promote NF-*κ*B pathway activation, in turn leading to GBP induction and the inhibition of caspase-3 activation, thus preventing macrophages from undergoing apoptotic death such that *Mtb* can thrive and replicate within these host cells [30]. Multiple reports have shown *Mtb* infection to be linked to the upregulation of a range of GBPs including GBP1-7, with elevated levels of these genes being linked to the risk of disease progression [31, 32].

ATF3 (activating transcription factor 3) is an ATF/cAMP response element-binding transcription factor that can reportedly modulate the upregulation of proinflammatory cytokines through binding to the NF-*κ*B p65 subunit [33]. Significant ATF3 upregulation occurs in the context of *Mtb* infection through an early growth response 1- (EGR1-) dependent mechanism, while ATF3 knockdown increases bacterial titers within infected macrophages [34]. Functionally, ATF3 can activate the expression of inflammation-related genes including IL-6, IL-12, and TNF-*α* [34, 35]. In addition, ATF3 is a type I IFN-inducible gene, constituting a key component of an IFN negative feedback loop as a result of increasing levels of IFN-I in ATB [36].

There are certain limitations to this study. For one, while efforts were made to identify all relevant publically available datasets, the sample size for these analyses was relatively limited, potentially limiting the accuracy of these findings. Second, it is important to note that the hub genes identified in this study were only correlated with immune cells, and the available data are insufficient to support a causative relationship. Similarly, these hub genes were correlated with *Mtb* infection status, highlighting a need for further research to validate these findings. Third, while several DEGs were identified when comparing ATB and LTBI samples, these genes may not be specifically related to *Mtb* infection. For example, the overexpression of GBP1 significantly inhibits Kaposi's sarcoma-associated herpesvirus infection [37] and *Chlamydia trachomatis* [38]. Lastly, microarrays are associated with certain drawbacks (e.g., not a whole genome analysis, high levels of background signal, not qualitative and quantitative, and an inability to detect alternative splicing). Additional *in vitro* and *in vivo* analyses exploring the function of these hub genes will be vital to establish the mechanisms underlying the pathogenesis of *Mtb* infection.

In summary, the five hub genes identified in this study may be associated with the immunopathogenesis of *Mtb* infections and offer potential utility as biomarkers that can be used to differentiate between ATB and LTBI patients.

## Data Availability

Publicly available datasets were analyzed in this study. These data can be found in GSE19491(https://www.ncbi.nlm.nih.gov/geo/query/acc.cgi?acc=GSE19491), GSE98461(https://www.ncbi.nlm.nih.gov/geo/query/acc.cgi?acc=GSE98461), and GSE152532 (https://www.ncbi.nlm.nih.gov/geo/query/acc.cgi?).

## Supplementary Materials

Supplementary 1 Supplementary file 1: details of GO analyses.

Supplementary 2 Supplementary file 2: details of KEGG analyses.

## References

[1] Gong W., Liang Y., Wu X. (2018). The current status, challenges, and future developments of new tuberculosis vaccines. *Human Vaccines & Immunotherapeutics*.

[2] Harding E. (2020). WHO global progress report on tuberculosis elimination. *The Lancet Respiratory Medicine*.

[3] Churchyard G., Kim P., Shah N. S. (2017). What we know about tuberculosis transmission: an overview. *The Journal of Infectious Diseases*.

[4] Floyd K., Glaziou P., Zumla A., Raviglione M. (2018). The global tuberculosis epidemic and progress in care, prevention, and research: an overview in year 3 of the end TB era. *The Lancet Respiratory Medicine*.

[5] Pai M., Behr M. (2016). Latent *Mycobacterium tuberculosis* infection and interferon-gamma release assays. *Microbiology spectrum*.

[6] Gualano G., Mencarini P., Lauria F. N. (2019). Tuberculin skin test - outdated or still useful for latent TB infection screening?. *International Journal of Infectious Diseases*.

[7] Zou J., Wang E. (2019). Cancer biomarker discovery for precision medicine: new progress. *Current Medicinal Chemistry*.

[8] Ibrahim B., McMahon D. P., Hufsky F. (2018). A new era of virus bioinformatics. *Virus Research*.

[9] Coppola M., Ottenhoff T. H. (2018). Genome wide approaches discover novel Mycobacterium tuberculosis antigens as correlates of infection, disease, immunity and targets for vaccination. *Seminars in Immunology*.

[10] Cai Y., Dai Y., Wang Y. (2020). Single-cell transcriptomics of blood reveals a natural killer cell subset depletion in tuberculosis. *eBioMedicine*.

[11] Martínez-Pérez A., Igea A., Estévez O. (2021). Changes in the immune phenotype and gene expression profile driven by a novel tuberculosis nanovaccine: short and long-term post-immunization. *Frontiers in Immunology*.

[12] Hu G., Grover C. E., Arick M. A., Liu M., Peterson D. G., Wendel J. F. (2021). Homoeologous gene expression and co-expression network analyses and evolutionary inference in allopolyploids. *Briefings in Bioinformatics*.

[13] Berry M. P., Graham C. M., McNab F. W. (2010). An interferon-inducible neutrophil-driven blood transcriptional signature in human tuberculosis. *Nature*.

[14] Sibley L., Gooch K., Wareham A. (2019). Differences in monocyte: lymphocyte ratio and tuberculosis disease progression in genetically distinct populations of macaques. *Scientific Reports*.

[15] Rakotosamimanana N., Richard V., Raharimanga V. (2015). Biomarkers for risk of developing active tuberculosis in contacts of TB patients: a prospective cohort study. *The European Respiratory Journal*.

[16] Joosten S. A., Fletcher H. A., Ottenhoff T. H. (2013). A helicopter perspective on TB biomarkers: pathway and process based analysis of gene expression data provides new insight into TB pathogenesis. *PLoS One*.

[17] Mayer-Barber K. D., Barber D. L. (2015). Innate and adaptive cellular immune responses to *Mycobacterium tuberculosis* infection. *Cold Spring Harbor perspectives in medicine*.

[18] Harari A., Rozot V., Enders F. B. (2011). Dominant TNF-*α*^+^*Mycobacterium tuberculosis*-specific CD4^+^ T cell responses discriminate between latent infection and active disease. *Nature Medicine*.

[19] Lindestam Arlehamn C. S., Lewinsohn D., Sette A., Lewinsohn D. (2014). Antigens for CD4 and CD8 T cells in tuberculosis. *Cold Spring Harbor Perspectives in Medicine*.

[20] Cadena A. M., Fortune S. M., Flynn J. L. (2017). Heterogeneity in tuberculosis. *Nature Reviews. Immunology*.

[21] Achkar J. M., Chan J., Casadevall A. (2015). B cells and antibodies in the defense against *Mycobacterium tuberculosis* infection. *Immunological Reviews*.

[22] Tran A. C., Kim M. Y., Reljic R. (2019). Emerging themes for the role of antibodies in tuberculosis. *Immune Network*.

[23] Basaraba R. J., Hunter R. L. (2017). Pathology of tuberculosis: how the pathology of human tuberculosis informs and directs animal models. *Microbiology Spectrum*.

[24] Jin J., Cardozo T., Lovering R. C., Elledge S. J., Pagano M., Harper J. W. (2004). Systematic analysis and nomenclature of mammalian F-box proteins. *Genes & Development*.

[25] Du X., Meng F., Peng D. (2019). Noncanonical role of FBXO6 in regulating antiviral immunity. *Journal of Immunology*.

[26] Decker T., Muller M., Stockinger S. (2005). The yin and yang of type I interferon activity in bacterial infection. *Nature Reviews. Immunology*.

[27] Manca C., Tsenova L., Freeman S. (2005). Hypervirulent *M. tuberculosis* W/Beijing strains upregulate type I IFNs and increase expression of negative regulators of the Jak-Stat pathway. *Journal of Interferon & Cytokine Research*.

[28] Eum S. Y., Kong J. H., Hong M. S. (2010). Neutrophils are the predominant infected phagocytic cells in the airways of patients with active pulmonary TB. *Chest*.

[29] Tretina K., Park E. S., Maminska A., MacMicking J. D. (2019). Interferon-induced guanylate-binding proteins: guardians of host defense in health and disease. *The Journal of Experimental Medicine*.

[30] Kim B. H., Shenoy A. R., Kumar P., Das R., Tiwari S., MacMicking J. D. (2011). A family of IFN-*γ*–inducible 65-kD GTPases protects against bacterial infection. *Science*.

[31] Yao X., Liu W., Li X. (2022). Whole blood GBP5 protein levels in patients with and without active tuberculosis. *BMC Infectious Diseases*.

[32] Chen J., Liu C., Liang T. (2021). Comprehensive analyses of potential key genes in active tuberculosis: a systematic review. *Medicine (Baltimore)*.

[33] Ku H. C., Cheng C. F. (2020). Master regulator activating transcription factor 3 (ATF3) in metabolic homeostasis and cancer. *Frontiers in Endocrinology (Lausanne)*.

[34] Kumar M., Majumder D., Mal S. (2020). Activating transcription factor 3 modulates the macrophage immune response to *Mycobacterium tuberculosis* infection via reciprocal regulation of inflammatory genes and lipid body formation. *Cellular Microbiology*.

[35] Thompson M. R., Xu D., Williams B. R. (2009). ATF3 transcription factor and its emerging roles in immunity and cancer. *Journal of Molecular Medicine (Berlin, Germany)*.

[36] Labzin L. I., Schmidt S. V., Masters S. L. (2015). ATF3 is a key regulator of macrophage IFN responses. *Journal of Immunology*.

[37] Zou Z., Meng Z., Ma C., Liang D., Sun R., Lan K. (2017). Guanylate-binding protein 1 inhibits nuclear delivery of Kaposi’s sarcoma-associated herpesvirus virions by disrupting formation of actin filament. *Journal of Virology*.

[38] Di Pietro M., Filardo S., Frasca F. (2020). Interferon-*γ* possesses anti-microbial and immunomodulatory activity on a *chlamydia trachomatis* infection model of primary human synovial fibroblasts. *Microorganisms*.

